# Identification of representative species-specific genes for abundance measurements

**DOI:** 10.1093/bioadv/vbad060

**Published:** 2023-05-08

**Authors:** Trine Zachariasen, Anders Østergaard Petersen, Asker Brejnrod, Gisle Alberg Vestergaard, Aron Eklund, Henrik Bjørn Nielsen

**Affiliations:** Department of Health and Technology, Technical University of Denmark, Lyngby 2800, Denmark; Department of Health and Technology, Technical University of Denmark, Lyngby 2800, Denmark; Department of Health and Technology, Technical University of Denmark, Lyngby 2800, Denmark; Department of Health and Technology, Technical University of Denmark, Lyngby 2800, Denmark; Clinical Microbiomics A/S, Copenhagen 2100, Denmark; Clinical Microbiomics A/S, Copenhagen 2100, Denmark

## Abstract

**Motivation:**

Metagenomic binning facilitates the reconstruction of genomes and identification of Metagenomic Species Pan-genomes or Metagenomic Assembled Genomes. We propose a method for identifying a set of *de novo* representative genes, termed signature genes, which can be used to measure the relative abundance and used as markers of each metagenomic species with high accuracy.

**Results:**

An initial set of the 100 genes that correlate with the median gene abundance profile of the entity is selected. A variant of the coupon collector’s problem was utilized to evaluate the probability of identifying a certain number of unique genes in a sample. This allows us to reject the abundance measurements of strains exhibiting a significantly skewed gene representation. A rank-based negative binomial model is employed to assess the performance of different gene sets across a large set of samples, facilitating identification of an optimal signature gene set for the entity. When benchmarked the method on a synthetic gene catalog, our optimized signature gene sets estimate relative abundance significantly closer to the true relative abundance compared to the starting gene sets extracted from the metagenomic species. The method was able to replicate results from a study with real data and identify around three times as many metagenomic entities.

**Availability and implementation:**

The code used for the analysis is available on GitHub: https://github.com/trinezac/SG_optimization.

**Supplementary information:**

Supplementary data are available at *Bioinformatics Advances* online.

## 1 Introduction

Metagenomic binning tools, such as MetaBAT 2 (Kang *et al.*, 2019), VAMB (Nissen *et al.*, 2021) and MSPminer (Plaza Oñate *et al.*, 2019), facilitate the reconstruction of genomes and identification of metagenomic entities, such as Metagenomic Species Pan-genomes (MSPs) or Metagenomic Assembled Genomes (MAGs), by gathering groups of genetic components, such as genes or contigs, that are believed to originate from a clade. The clade is typically at the species or subspecies level, where the gene composition is relatively conserved (Nielsen *et al.*, 2014). The composition of a typical metagenomic sample is a priori unknown and may contain novel organisms, new variants of already characterized organisms, and closely related but distinct organisms. This challenges the metagenomic detection and quantification of the microbiome, since sequence reads from one species may map perfectly to the reference sequences of another species (Sangwan *et al.*, 2016). Stringent mapping may reduce the cross-species mapping, but this may come at the expense of robustness in quantifying variants of a species. Genes that are specific to a given strain, yet present in all members of that clade, are ideally suited for measuring the abundance of the species by eliminating cross-mapping of reads while allowing for accurate and precise measure of a given strain. Additionally, SGs should not be duplicated within a strain to avoid biasing the abundances of strains whose genes have high copy numbers. Such a set of genes is referred to as a signature gene set (Segata *et al.*, 2012). SGs have previously been identified by comparing reference genomes from species-level clades (Milanese *et al.*, 2019). This approach works when sufficiently many reference sequences are available from a given species as well as from the species from which the reference is to be distinguished from. However, for species with few available reference genomes, or few genomes from related species, it is difficult to define signature gene sets. Additionally, certain genomic sequences are easier to sequence, yielding more reads and a skewed read distribution throughout the genome. A selection of signature genes that does not account for this has the potential to artificially inflate the relative abundance of certain species. When no references are available, a set of SGs can be identified based on their ability to quantify a species (or any clade of interest) in a given context, e.g. the human microbiome. We propose a method that relies entirely on a statistical analysis of the distributions of readmappings to the genes and that is entirely agnostic to bias in read generation, gene duplication, etc. This method searches for gene sets that produce robust and even mapping across natural population variability and minimize signal noise. Within each sample, the expected number of mapped reads per gene can be approximated by the discrete negative binomial (NB) distribution (Zhang *et al.*, 2017), as the reads are assumed to map in proportion to the gene length and exhibit some degree of variability. As the gene lengths are known, the total number of sequence reads that map to a good signature gene set should predict the number of genes in the set that the reads map to. In other words, the reads should appear to be drawn randomly from across the gene set. Large deviance from the expected model could be due to violations of the aforementioned characteristics of a good signature gene, i.e. genes that are not omnipresent to a given strain or not present in all members of that strain. Here, we illustrate the necessity for such an approach and propose a method for defining optimal gene sets and for estimating the likelihood that the observed read mappings only originate from a population that comprised the complete SG set in equal quantities. In this article, we propose (i) a method for selecting optimal signature gene sets and (ii) the use of a special case of the ‘coupon collector’s problem’ (CCP) to assess the likelihood that sequence reads will map to a specific number of genes (*d*) given the number of reads (*k*) that map to the entire gene set.

Binning is typically divided into two major approaches, gene based and contig based. Contig-based binning is especially useful when trying to reconstruct whole genomes, while gene-based binning is useful for identifying and characterizing microbial communities at a higher taxonomic level. The method has been created to aid in *de novo* identification of species, for both gene- and contig-based methods as well as for profiling of species defined by reference genomes. The following results stem from the analysis of a simulated gene catalog (SGC) from Borderes *et al.* (2021) as well as a case study performed on the First Year of Life Dataset from Bäckhed *et al.* (2015).

## 2 Methods

### 2.1 Input data and formatting

The input for our method is a gene count matrix (comprising information about the number of mapped reads to each gene within each sample) as well as information linking the genes to their corresponding biological entity. In this study, we illustrate two different methods for creating these data structures.

#### 2.1.1 The SGC

The non-redundant SGC used in this study is meticulously designed by Borderes *et al.* (2021). The short reads of the SGC are created by GenSIM (v.1.6) (McElroy *et al.*, 2012) and constructed based on the genomes of 47 strains belonging to 41 species and theoretical abundance profiles of 40 samples. Borderes et al. mapped the reads using MOCAT (v.2.0) (Kultima *et al.*, 2012) and SOAPALIGNER2 (Li *et al.*, 2009) with the ‘allbest’ mapping mode, and to generate gene abundance profiles for all samples (Kultima et al., 2012; Li et al., 2009). As the genes of the species and their corresponding abundances are known in the SGC, a golden standard has been created containing the gene identifiers and their associated species.

#### 2.1.2 The MSPs

MSPminer (v. updated 2018-04-25) (Plaza Oñate *et al.*, 2019) has been applied to the SGC to identify the MSPs. Each MSP is a collection of clustered genes belonging to a biological entity. MSPminer is run with default parameters and the results are summarized in a tab-separated file, containing the genes and its corresponding MSPid. MSPminer divides the reads into a total of 54 bins with the number of clustered genes ranging from 575 to 5957. Each MSP is on average present in 36 samples, ranging from a minimum of 26 samples to a maximum of 40.

#### 2.1.3 A case study using Bäkhed's First Year of Life dataset

The First Year of Life study has been used as a case study, to illustrate the SG method in a well-designed study with high-quality data. The dataset constructed by Bäckhed *et al.* (2015) comprises a total of 392 short-read samples from 98 infants [3 different timepoints (Newborn, 4M, 12M) and a sample from their mother]. The samples have been shotgun sequenced with an average of 3.99 Gb of reads per sample.

#### 2.1.4 Creation of VAMB clusters

The samples have been through preprocessing including adapter removal using BBDuk (v. 38.96 http://jgi.doe.gov/data-and-tools/bb-tools/), removal of low-quality reads and reads shorter than 75 base pairs using Sickle (v. 1.33) (Joshi and Fass, 2011) and removal of human contamination (reference UCSC version hg19, GRCh37.p13) with BBmap (http://jgi.doe.gov/data-and-tools/bb-tools/).

*De novo* assembly was carried out per sample with Spades (v. 3.15.5) run with the meta-option (15) and kmer sizes of 213 355 and 77. Contigs <1500 bp were discarded. BWA-mem2 (v.2.2.1) (Vasimuddin *et al.*, 2019) and SAMTOOLS (v.1.10) (17) were used for mapping the reads to the assemblies. Metabat2’s jgi_summarize_bam_contig_depths (v.2.12) (Kang *et al.*, 2019) was used to assess the depths of the contigs. VAMB (v.3.0.8) (Nissen *et al.*, 2021) was run with default parameters to bin the contigs. Annotation of the bins along with gene predictions was done using GTDK-tk (v.2.1.1) (Chaumeil *et al.*, 2022). The genes were clustered using MMseqs2 (v. 13.45111) (Steinegger and Söding, 2017) with a sequence identity threshold for the clustering of genes of 0.8. The remaining genes are used for the construction of a gene count matrix for each VAMB cluster, containing the read counts of each gene within each sample.

### 2.2 Data preparation

The statistical analysis and data handling have been performed in R (v.4.1.2) (R Core Team, 2021). The genes of the entity are sorted according to co-abundance with the genes with highest intra-species abundance correlation as the first genes within the entity. Different sizes of gene sets were evaluated by comparing the absolute difference in abundance between the predicted and the true abundance using the SGC. We tested the gene set sizes in the range between 70 and 150 and found a local minimum of 100 genes. A metagenomic species is considered detected in a sample if it contains reads that map to three or more signature genes. The read counts, which are normalized according to gene length, are multiplied by 1000 to avoid small numbers and rounded to the closest integer.

### 2.3 Development of benchmark

To assess the chance of identifying *d* out of *n* SGs given *k* reads assigned to signature genes, we use an analytical solution to a variant of the CCP described in a 2008 conference summary published from the *27th International Conference on Technology in Collegiate Mathematics*, by Fowler (2015). The variant of the CCP tackled in this article is the chance of drawing exactly *d* different balls out of an urn containing *n* different balls given *k* draws of one ball with replacement. The solution to this problem is


(1) $$P\left(d,k,n\right)=\frac{\left(\frac{n}{d}\right)}{n^{k}}S\left(k,d\right)d!$$

As the generation of Stirling numbers of the second kind *S*(*k*, *d*) is computationally intensive, pre-computed values of *S*(*k*, *d*) are obtained from www.planetcalc.com, a resource for solutions to common mathematical problems. This resource lists solutions for *k* = {0, 1, 2, **…** 176}, *d* = {0, 1, 2, **…***k*}. Eighty-six percent of *P*-values encountered in the dataset can be computed in this manner for *n* = 100. For the estimation of *P*(*d*, *k*, *n*) in cases where *k* > 176 and/or *n^k^* is evaluated as Inf by R, utilization of pre-computed bootstrapping results is carried out. Bootstrapping is carried out by randomly sampling *n* genes *k* times and evaluating the number of different genes obtained, *d*, 10^5^ times. The chance of obtaining exactly *d* out of *n* genes given *k* reads is evaluated as the number of times *d* unique genes was obtained out of 10^5^ iterations. Bootstrapping is carried out for *k* = {0, 1, 2, **…** 3000}, to *n* = {0, 1, 2, **…***k*} for a given value of *n*. Solutions to *P*(*d*, *k*, *n*) where *k* > 3000 are thus approximated as and


(2) $$P\left(d=n,k,n\right)∼1,\;k\geq 3000$$

(3) $$P\left(d<n,k,n\right)∼0,\;k\geq 3000,\;n\geq 3000$$

To assess the degree of accuracy of bootstrapping, *P*-values obtained by bootstrapping were compared to *P*-values obtained by analytical evaluation across the entire dataset tested, the Pearson correlation coefficient (PCC) was evaluated as 0.99. Thus, this variation in the CCP is made to evaluate the performance of a set of SGs as a whole, ensuring that none of the genes are disproportionately easy or difficult to identify.

### 2.4 Signature gene refinement

#### 2.4.1 Introduction

**Fig. 1.**
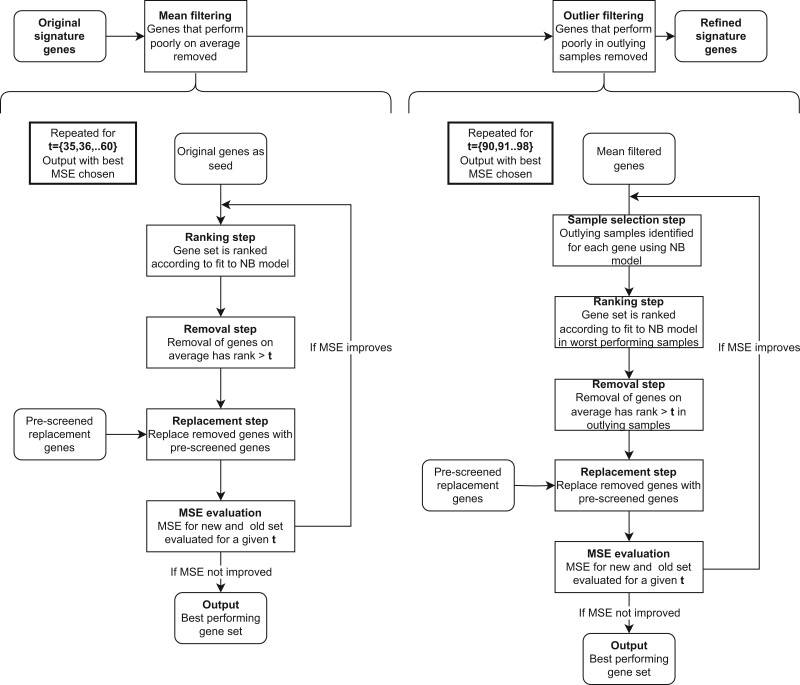
Two-step signature gene refinement algorithm as described in methods. NB: negative binomial

The performance of individual genes is evaluated using an NB model, which evaluates whether higher sequencing depth also reliably leads to higher read counts. The ranking of the genes enables the detection and removal of SG, which are found inconsistently across the samples. As the method utilizes the power across samples, one limitation to the method is that it requires at least three samples. Genes are ranked according to how well they fit this model in each sample and replaced with genes evaluated with CCP. Initially, all samples that contain three or more reads towards a certain set of SGs are identified. In the first step, SGs are exchanged if their mean rank across samples is above a certain threshold, *t*. In this way, we only replace genes that consistently underperform across multiple samples. Genes are ranked by how well they fit the NB model in each sample by the size of the residual, and the mean is taken across samples resulting in the mean rank. If the exchanged SG set has a lower mean squared error (MSE) than the previous set, the SG set is kept and reruns until the MSE no longer decreases. The method is repeated, this time assessing whether any genes are outlying in a subset of the samples. If the MSE improves for multiple thresholds, the refined SG set is selected as the one with the lowest MSE (Fig. 1).

#### 2.4.2 Frequency-based filtering

The ranking of genes using an NB model ensures removal of genes from the original SG set whose detection is inconsistent across samples. However, it does not ensure that selected genes have a similar ease of detection across samples. Ideally, the genes within an SG set are found with an equal probability; however, it is expected that biological and technical bias will lead to a skewed sampling of the genes. This can lead to systemic biases in abundance estimation that favour the abundances of strains with sensitive SGs. Additionally, a good set of SGs should be sensitive, i.e. be part of genomic sequences that are easily sequenced, to detect low-abundant strains in a sample. To accommodate this prior to the refinement of the SGs, the genes need to be prescreened and ordered according to their sensitivity, such that an increase in *k* will entail an increase in *d* for the samples. The over- or undersampling of genes is alleviated using systematic replacement of genes, implementing a pre-filtering step in which a set of SGs with similar, high sensitivities were selected for replacing poorly performing genes, while avoiding genes whose sensitivities were very different from the other SGs. The genes assigned to the respective metagenomic entity are sorted in order of decreasing frequency of detection across all samples. A set of 700 genes are selected, which have the highest overall frequency of detection, excluding genes whose frequencies were outside the 1.2 interquartile range of the rest of the set. Thereby selecting genes with a high frequency of detection, but at the same time are also found in a consistent manner, ensuring the SGs that are used for replacement are all easy to detect. The genes used for the replacement of the SGs are found within this pool of 700 genes leading to a more heterogeneous frequency of detection of the genes included in the final SG set. In the case of an entity with <700 genes, all genes are used.

#### 2.4.3 Ranking of genes

As part of identification of genes that should be removed, first, we must evaluate genes based on the consistency of detection. To assess the performances of each gene, an NB distribution is used to test whether increased sequencing depth reliably leads to additional counts of that gene. How consistently a gene is detected has previously been shown to follow an NB distribution (Zhang *et al.*, 2017) as the NB model is known to handle overdispersion that is frequently observed in sequencing data. The mentioned model is applied for each sample, where the read count of gene *i* in the *j*th sample is denoted $y_{ij}$, then where $\mu_{j}$ is the average read count per gene, $\sigma_{j}$ is the sample-specific NB dispersion parameter and Γ (·) denotes the gamma function. The NB model can be seen as a compounded Poisson-Gamma distribution, in which the rate parameter of the Poisson model itself is a random variable distributed according to a Gamma distribution (Zhang *et al.*, 2017). When the distribution approaches the Poisson distribution with equal mean and variance. From this parametrization of the NB model, the expected read count is given as $E\left[y_{ij}\right]=\mu_{j}=\lambda_{ij}N_{j}$, where $\lambda_{ij}$ is the proportion of reads mapped to gene *i* in the *j*th sample and $N_{j}$ is the total number of reads mapped to sample *j*; thus, $\mu_{j}$ depends on the sequencing depth as well as the abundance of the species in the sample. The variance of the read count is given as $var\left(y_{ij}\right)=\mu_{j}+{\mu_{j}}^{2}1/\sigma_{j}$. The counts of each SG are evaluated according to this NB model and are ranked within each sample by evaluating the difference between the expected count and observed count.


(4) $$y_{ij}∼NB\left(\;y_{ij}|\mu_{j},\sigma_{j}\right)=\Gamma \left(y_{ij}+\sigma_{j}\right)/\Gamma \left(\sigma_{j}\right)y_{ij}!\cdot {\left(\sigma_{j}/\mu_{j}+\sigma \right)}^{\sigma_{j}}\cdot {\left(\mu_{j}/\mu_{j}+\sigma_{j}\right)}^{y_{ij}},\;\mu_{j}>0$$

#### 2.4.4 Rejection and replacement

We use the mean rank of each gene across the samples to evaluate the performance of the SG, which enables the detection of SG with persistent discrepancies according to the NB model. If the genes have a lower average rank than a given threshold, consequently underperforming, the genes will be removed from the SG set, thereby leaving a smaller SG set, in which we have higher confidence that the genes are consistently found across samples. A range of thresholds are tested to obtain the best possible gene set. If the remaining SG set maps to <10 samples, the refined SG set will not be considered for further analysis, as the data are too scarce for reliably ranking of the SGs. The NB model is reapplied to the retained SG set to exclude potential noise caused by the already removed genes. The NB distribution is fitted exclusively on the genes, which we believe to reliably lead to an increase in SG detection as sequencing depth increases. A subset of the frequency-based filtered pool of genes are introduced to the SG set, leaving a complete SG set of 100 genes. The introduced subset is selected as the genes with the highest co-abundance, which were also accepted in the filtering step and have not already been included in the SG set. When assuming that each read has an equal change of mapping to each signature gene and that the mapping process of each read is independent of the previous reads, the probability of a gene not being detected can be described by


(5) $$P_{0}=\frac{{\left(n-1\right)}^{k}}{n}$$

where *n* is the number of signature genes and *k* is the number of reads that map to the SGs in that sample. By taking the complement of *P_0_*, we can calculate the probability of an SG being detected can thus be calculated as $P_{1}=1-P_{0}$. This can be utilized to calculate the expected number of detected signature genes *d*, which for each sample *j* as


(6) $$d_{j}=\left(1-{\left(n-1\right)}^{k_{j}}/n\right)n,\;j=1,2,\ldots ,m$$

where $k_{j}$ is the total number of reads mapped to the SGs and *m* is the number of samples. We assume that each read has an equal chance of mapping to each signature gene (after gene length normalization) and that the mapping process of each read is independent of the previous reads. The effect of SG replacement is evaluated based on the deviation from the expected distribution [Equation (5)]. Only if the deviation has been reduced, the changes to the SG are kept. The process of ranking, removing and replacing is repeated until the MSE is reduced by less than 1% from the previous iteration. The result is kept for the threshold that performs the best. By iteratively improving the SG set and reevaluating the NB approximation the gene set are continuously improved, leading to a gene set that is more reliable for abundance estimation of the species, as the genes are more often present within the majority of the strains.

The optimum threshold varies between the metagenomic entities, as each set of SG deviates from the expected distribution [Equation (5)] differently, leading to a different spread of the mean rankings of the SG. If the SGs are detected consistently across the samples, the SGs will have a small spread in average rank. In case the detection of the SGs is inconsistent, a large spread in the average ranking of the genes will be observed. In the first step, referred to as mean filtering, all integers in the range 35–60 are tested to identify the threshold for mean-rank leading to the genes, which are most reliably detected across samples to accommodate the differences of the metagenomic entities. The range of thresholds is selected, as testing indicates that the majority of optimal thresholds for obtaining the best possible SG set falls well within this range.

In some cases, one or more genes will be consistently missing in a smaller subset of samples, which the mean-based filtering across all samples cannot alleviate. To capture these genes, the ranking method is re-implemented, but where the average ranking of the SG was previously used, we are now evaluating the genes based on the 95th percentile according to adherence to the expected distribution [Equation (5)]. By selecting the 95th percentile, we are considering only the 5% of the samples that perform the worst. If SGs are persistently diverging from the NB model in this subset of samples, they are tried and replaced after which it is examined whether the refined SG set follows the expected distribution more closely. The optimal threshold for the removal of SGs based on the rank of the 95th percentile is found in the range 90–98.

### 2.5 Benchmark calibration

The goal of identification of SGs is to obtain a sizable set of genes that are shared by all members of a strain. Ideally, all 100 SGs are identified in all samples with high sequencing depth; however, it is very difficult to select a set of SGs that are all identified synchronistically when many reads are assigned to SGs, indicated by high *k* values. Any sample with very high read depth that contains the metagenomic entity at an adequate abundance would result in a *k* that approaches the number of SGs chosen for that strain and at very high sequencing depths the chance of not finding all *n* SGs approaches zero. Any biological variation in SGs in deeply sequenced samples would be rejected and assigned a very low probability of occurring due to the assumption that all SGs are present without biological variation. This limits the applicability of this method. To allow for a biologically unsubstantial degree of variation in SGs, we will consider the chances of obtaining *d* or fewer unique SGs out if a species contains *at least n* SGs given *k* assigned reads instead of all *n*. We must arrive at a value of *n* that does not unfairly reject samples that are missing an inconsequential amount of SGs due to unimportant degrees of biological variation, while still rejecting samples whose metagenomic entity shows a clear lack of SGs, e.g. due to strain differences. A fair threshold should be able to distinguish samples in which strain differences show from the SGs but still allow for smaller biological differences to appear. We select *n *=* *95, such that for a random distribution, one would expect approximately 5% of the samples to fall below *P *<* *0.05. MSP07 is the best-performing initial gene set in our catalog with an MSE of 0.7 and setting an *n* of 95 rejects 1/40 of samples, which we consider to be a sufficient approximation. The null hypothesis we test against is that a sample contains 95 different SGs with an equal chance of finding each SG. The choice of rejecting or accepting a sample will be evaluated as the chance of obtaining *d* or fewer different SGs out of 95 SGs, given *k* reads assigned to SGs.

## 3 Results

### 3.1 Identifying the optimal signature gene sets

Typically, a strong relationship is observed between the number of reads mapped to the metagenomic entity and the number of detected genes within the gene set of each sample. However, for some MSPs, part of the initial gene set is rarely detected despite high coverage of the remaining genes. The gene set is selected as the 100 genes with the highest co-abundance correlation (Pearson’s correlation coefficient) to the median abundance for all genes. An NB model was used to assess the probability that all of the genes in a given gene set are present, in exactly one copy per genome, in a sample, given the observed read mappings. The probability that a sample contains the complete gene set is dependent on the number of reads that map to the gene set (*k*), the number of different genes from the SGs detected in the sample (*d*) and the threshold for what is considered a complete SG set (*n*). Using this statistical framework, we can evaluate the expected number of detected genes from the set, *d*, given a number of mapped reads, *k.* From this expected distribution, we can evaluate the performance of an SG set by its MSE between the observed and expected numbers of identified genes from the set across a series of samples. During refinement of the gene set, the MSE was used to reject changes to the SG set that led to an increase and accept changes that reduced it. During the evaluation of individual samples, some biological variation is allowed by setting the *n* to 95; hence, a species, with a given SG, is considered detected in a sample if the observed reads mapped fit with an SG set with at least 95 genes. When evaluating MSEs, for refinement or otherwise, the expected distribution is derived from a distribution with *n *=* *100 to avoid optimizing for an incomplete set of SGs.

Refinement was done using a two-step approach as described in methods, which relies on replacement of genes that perform consistently poorly across multiple samples. Poor performing genes were replaced until improvement of MSE was negligible. The performance of the improved signature gene sets will be assessed on four parameters: model fit (MSE), amount of initial SGs retained, PCC of counts between the initial and refined SG sets and change in the number of samples where the observed reads mapped fit with significantly reduced number of genes in the SG. To allow for a negligible amount of biologically variation in SG evaluation, samples thought to contain 95 or more different SGs will be accepted as wholly.

**Fig. 2.**
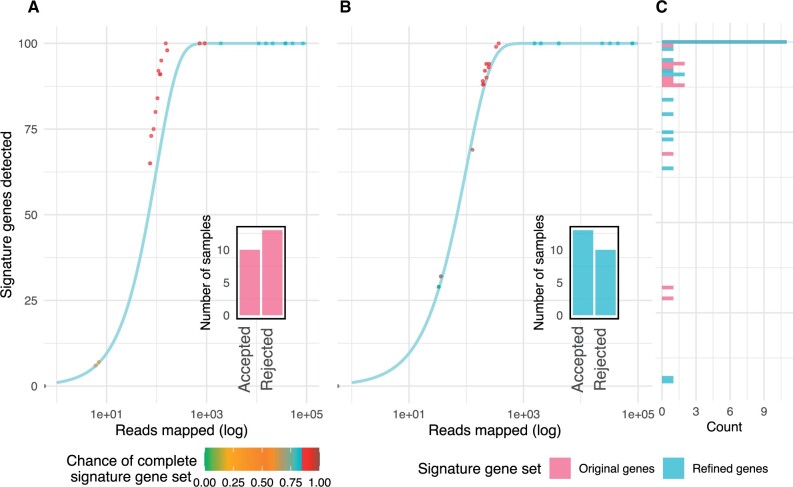
Detection of signature genes. (**A** and **B**) Distributions of the number of different identified signature genes for a given number of reads mapped to signature genes for each sample for MSP54. Colours indicate the chance of this sample containing 95 unique signature genes as described in methods. The bar plot indicates the number of samples that were rejected (*P *<* *0.05, CCP) and accepted (*P *>* *0.05, CCP). The expected distribution of samples for a metagenomic entity which contains 100 SGs is indicated by a blue line. Panels (A) and (B) are for SGs prior to and after SG refinement, respectively. Panel (**C**) indicates the distribution of uniquely identified SGs across samples, red indicating pre-refinement and blue post-refinement

The method was applied to the SGC created from 40 simulated metagenomics samples with reads from 47 reference strains (Borderes *et al.*, 2021). To assess how close a set of SGs follow the expected distribution, the MSE between the observed and expected numbers of different SGs was evaluated for the pre- and post-refinement SG sets. To clearly illustrate the issues with original signature gene sets and the changes occurring in each species between pre- and post-refinement SGs, *Buchnera aphidicola* (MSP54) will be used to exemplify the changes, after which summary statistics will be given for the improvement of all MSPs (Fig. 2). *B.aphidicola* was chosen because the original SG set exhibits some of the issues we are addressing in this article, namely large amounts of samples with a shifted distribution, indicating a heterogeneous ease of detection of SGs. MSP54 initially exhibited an MSE of 110.57, which after refinement was reduced to 2.13, showing a set of SGs that follow the expected distribution much closer after refinement. The MSP is mapped with at least three reads in 23 samples. Prior to refinement, 10 of these samples were accepted (*P *>* *0.05, CCP), indicating that the amount of detected SGs is coherent to the number of reads mapped to the MSP. After refinement, 13 of the samples are accepted (*P *>* *0.05, CCP), indicating that the replacement genes are more compliant with the probabilistic model. Across all MSPs, the MSE was evaluated for the initial and refined SG sets. We observe a decrease in MSE for 28/54 MSPs (Supplementary Fig. S1). A significant lowering of MSE is observed between the initial SG set and the refined SG set by a Wilcoxon signed rank test (*P*-value of 4.0e−06 paired).

To assess the degree of SG exchange, the fraction of original SGs retained and the ratio of MSE before and after SG refinement were compared (Supplementary Fig. S2). In MSP54, 20 out of the 100 initial signature genes were retained; hence, a large proportion of the initial SGs were exchanged in favour of other genes. Across all MSPs, we observed a correlation between the relative MSE (MSE_before_ − MSE_after_) and the number of signature genes retained; however, no significant correlation was found. We observe the largest improvements in MSE in MSPs in which a large fraction of SGs have been replaced. The MSPs are having 38 genes replaced on average, with 18 of the MSPs replacing 75 or more of their SGs. Conversely, 10 MSPs change between 25 and 75 of its original SGs, while the remaining 26 MSPs experience no change in SGs.

A sample is rejected if the chance of obtaining *d* out of 95 signature genes given *k* reads assigned to signature genes is below 5% (*P *<* *0.05, CCP). Samples that contain fewer than three reads (*k *>* *3) assigned to the SGs were not considered as we are not confident in the detection of this metagenomic entity. Samples with fewer than three assigned reads were neither accepted nor rejected to avoid influence of samples that would otherwise not be considered for abundance measurement. For example, 23 samples were found to have three or more reads for MSP54 prior to refinement, 13 of which were significantly depleted in SGs. After refinement, 10 out of 23 samples with more than three reads assigned to SGs were significantly (*P *<* *0.05, CCP) depleted in SGs. Across all MSPs, prior to refinement, a significant depletion (*P *<* *0.05, CCP) in SGs was found in 18% of instances in which three or more of the initial SGs were mapped and were rejected. This rate is lowered after refinement, as 15% of instances were rejected.

Finally, we wish to evaluate the change in the number of samples with more than three reads assigned to SGs (*k *>* *3), which we consider to be an indicator that the organism is present in the sample. There were concerns that a reduction in MSE could be achieved by selection of a set of SGs that were exclusively found in a rare strain but not present in the vast majority of samples. To assess this, the degree of change in mapped samples (*k *>* *3) was evaluated. This number was correlated with the degree of change in MSE between initial and final SGs (Supplementary Fig. S3).

Of the 54 MSPs, only 11 of them display a change in number of mapped samples after the SG refinement. The average share of genes found across samples for the MSPs ranges from 0.69 to 0.99, indicating that once an MSP is identified within a sample, most of its SGs are detected and are thus estimated to be present in higher abundance. For MSP54, an average of 85 of the 100 initial SGs are identified in the samples, which are also seen in Figure 2C.

### 3.2 Relative abundance measures of the initial and refined signature genes

**Fig. 3.**
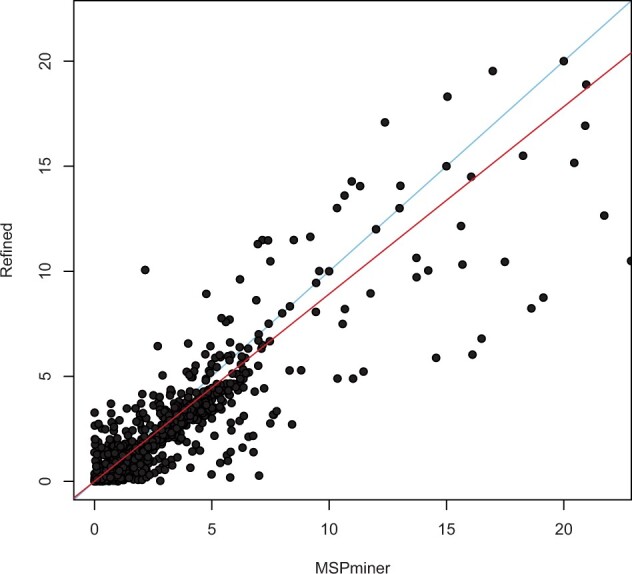
Error in relative abundance of species-level taxa. Error from true abundance predicted by MSPminer and true abundance to the refined SGs. The error is calculated as the absolute difference from the predicted relative abundance and the true relative abundance. Each dot represents a species-level taxa. The red line indicates the linear relationship between the two methods. The blue line indicates the identity line (*y* = *x*). For visualization purposes the seven taxa with the largest discrepancy from the true relative abundance has not been included on the figure

The sample-specific relative abundance is found by dividing the reads that map to the SGs by the total number of reads mapping to the SGs across the MSPs. MSPs with identical taxonomic annotations are collapsed into a single biological entity. The taxonomies are extracted per gene from the Golden Standard Single Assignment binning results from Borderes *et al.* (2021). If the SGs are assigned conflicting taxonomies, the most abundant taxonomy of the refined SG set is assigned to the MSP. Of the 47 genomes used to construct the SGC, 43 of the entities were represented by MSPs, yielding a precision of 0.81 and a recall of 0.91. The read counts of the genes are normalized according to the length of the genes. The error between the calculated relative abundances from the gene set of MSPminer and the refined SGs are computed (Fig. 3). The error in relative abundance tends to increase with an increase in the true relative abundance (Supplementary Fig. S4).

It was examined whether using the full gene set of the MSPs would yield better relative abundance estimates. When taking the reads that map to all of the genes comprised in the MSPs and comparing with the true relative abundance, the discrepancy is on average larger than for the initial SG set from MSPminer.

**Fig. 4.**
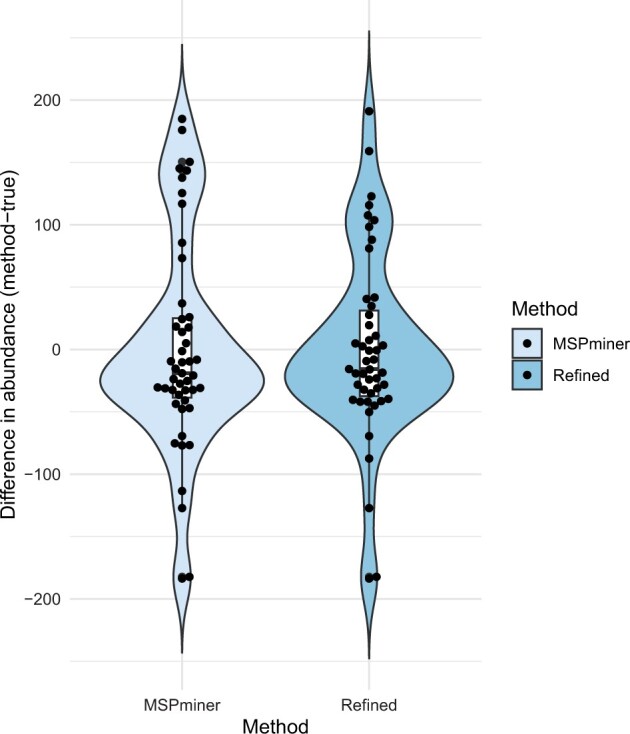
Difference in relative abundance for the truth subtracted the abundance given from the initial SGs from MSPminer and the refined SGs. Combination violin and boxplot of the biological entities

The relative abundance quantifications were compared with the true relative abundance by subtracting the true versus the calculated. The closer the abundance prediction is to the ground truth, the closer the value will be to zero. The differences in predicted and true abundance for both the initial SGs from MSPminer and the refined SGs the difference was evaluated (Fig. 4). The relative abundance of the refined SGs is found to be closest to the true abundance, when tested with a paired Wilcoxon signed rank test with a *P*-value of $2.2{\times 10}^{-16}$ and an effect size of 0.13. Especially the MSPs estimated by MSPminer to be in higher abundance than the ground truth are found in abundances closer to the ground truth after refinement.

### 3.3 Case study using Bäkhed's First Year of Life dataset

**Fig. 5.**
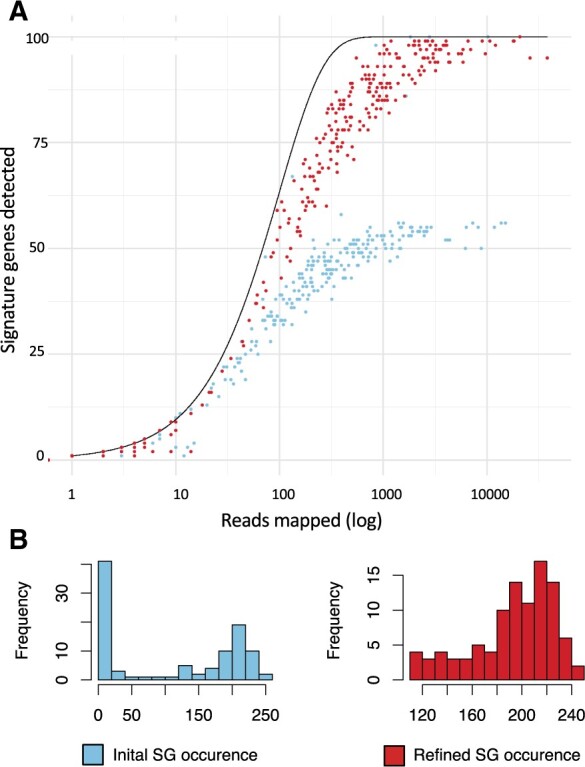
Read counts of Cluster5004 from the case study using data from Bäkhed et al. (**A**) Distributions of detected SGs displayed as a function of number of reads mapped to the SGs per sample for the initial SG found with VAMB and the refined SG set. The expected detection of SGs given by the number of mapped reads is indicated by a black line [Equation (6)]. (**B**) Histogram of the detection of each SG for the initial and the refined SGs

Bäkhed's dataset was used to demonstrate the applicability of SG in a real and well-structured metagenomics study. The study comprises samples obtained from infants and their mothers (Bäckhed *et al.*, 2015). The data were pre-processed followed by *de novo* assembly of contigs. Of the 392 samples, only 389 samples were successfully assembled after 20 days of runtime on our HPC (40 cores and 180 GB per job). The contigs were binned across samples with VAMB, and subsequent filtering (discarding bins <200 000 base pairs) resulted in 9763 bins from 2672 VAMB clusters. For reference, Bäkhed et al. identify 690 meta Operational Taxonomic Units (mOTUs). Further annotation of these clusters using GTDB-tk (v.2.1.1) was successful for 1843 clusters, where the original study annotated 373 species. Genes were predicted through GTDB-tk using Prodigal (v.2.6.3) (Hyatt *et al.*, 2010), 763 clusters were found in fewer than 3 samples or contained less than 100 genes and, consequently, the abundance was set to 0. For this dataset, an improvement in MSE between the SGs from VAMB and the refined SG’s is obtained for 587 of the 1080 clusters, with an average improvement of 42.6 ± 27.2%. Cluster5004 (annotated as Parabacteroides distasonis) is one of the clusters, which displays a large improvement in MSE (Fig. 5A), from an MSE of 984.17 to 121.05. In the initial SG set, 6 samples had above 61 detected signature genes despite a mean number of reads mapping across the samples of 7581 ± 10 851.56. The detection of the initial SGs is shown as a heatmap, where the genes and samples are grouped using hierarchical clustering (R Core Team, 2021) (Supplementary Fig. S5). The 6 samples with >61 genes detected cluster together to the left side of the figure. Fifty of the SGs are seen in less than 50 samples, despite the Cluster being present in 319 samples (Fig. 5B), with an average of detection of 111 reads per sample. For the refined SG, the average number of detections is 193 reads per sample (Fig. 5C).

**Fig. 6.**
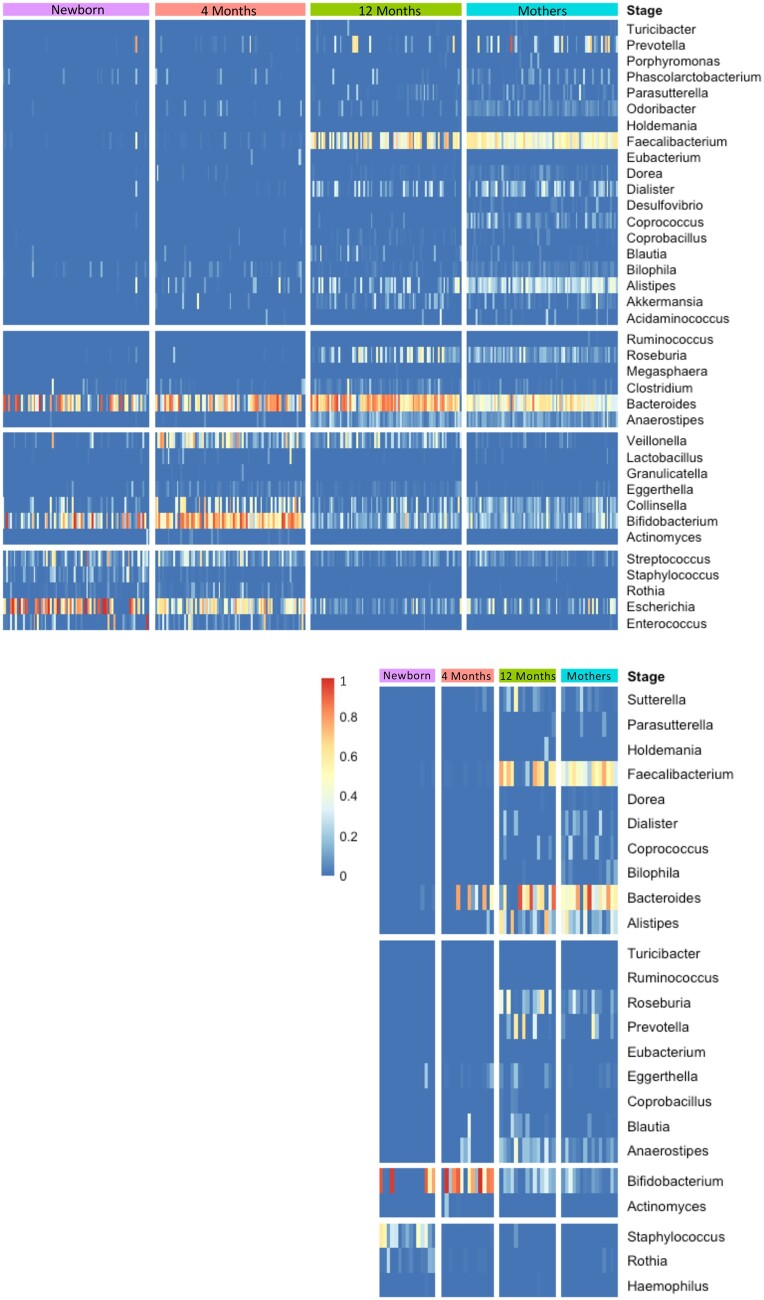
Heatmap of relative abundance of the Signature Genera found by Bäkhed et al. in infants born **(A**) vaginally or (**B**) by C-section at the stages newborn, 4 months, 12 months, and their mothers. Columns indicate samples. The vertical coloured blocks indicate Signature Genera at each stage

A beta-diversity analysis of the relative abundances was carried out using Bray–Curtis distances and visualized with a principal coordinate analysis plot (Supplementary Fig. S6).

To demonstrate the use of SG for abundance measurements, a replication of their *Signature Taxa* from each stage of the study was performed (Fig. 6). The taxa from their Supplementary Table S5 were used for the creation of the heatmap. Of the 57 Signature Genera found for vaginally born infants, 37 were reidentified and annotated. For the infants born with C-section, 24 of 37 Signature Genera was reidentified. Three additional Signature Genera were found in our results; however, GTDB-tk had them classified as ‘Family’ level (Erysipelotrichaceae, Lachnospiraceae and Ruminococcaceae).

## 4 Discussion

We utilized a variant of the CCP as a theoretical framework to implement SG refinement. The CCP appears to be a good approximation for the majority of refined SG sets, although certain SGs with heterogeneous sensitivities do not follow the initial assumptions of this method, as the CCP assumes uniform probabilities for sampling.

Despite the prescreening and ordering of SG according to sensitivity, we still find certain refined SG sets where samples appear to detect numbers of SGs deviating from the expected, given the number of mapped reads, especially samples that have had an inflated number of identified SGs. This is in accordance with the findings of Borderes *et al.* (2021), where MSPminer is found to overestimate the number of binned genes of the SGC. This could be due to the SGC representing a simplistic and not necessarily representative version of the human gut microbiome, being unable to capture nature's variability, leading to an underrepresentation of cross-mapping of reads between species and genes being mapped more often than expected. We successfully implemented the SG method on the First Year of Life study, where we were able to reconstruct and annotate 1843 clusters, compared to the 373 mOTUs found in the original study.

While the two-step refinement appears to be very good at detecting genes with low detection rates, outlying SGs with very high detection frequencies were not removed, which could contribute to this problem. This is to some extent alleviated by pre-filtering of genes and could potentially be further alleviated by starting with an alternate set of initial SGs. No explicit criteria were given to select genes that had similar sensitivities for initial SG sets, as this would narrow the number of genes that could be searched to an extent that could end up hampering MSE improvement and complicate the assessment of improvement between refinement steps. However, for Cluster5004 from The First Year of Life study, we were able to identify a suitable gene set, even when only 6 of the samples had >61 of the initial SGs detected. From the heatmap, it is clear that the six samples cluster and that there is a clear trend amongst which genes are consistently being detected. With half of the SGs having reads mapping in fewer than 50 out of 319 samples, this indicates that they are not part of the core genome of this cluster but are more likely strain specific. Or that the 6 samples having >61 detected SGs are part of a higher-level taxa than the rest. However, after the refinement, the SGs are detected more consistently across the samples.

In the absence of a large number of samples with adequate read counts, it is difficult to select suitable SGs for the metagenomic entity due to the fragmented nature of metagenomic count data. For rare species found in low abundance, the count data of the metagenomic entity will predominantly be zero inflated. The proposed model is not developed to explicitly deal with zero inflation. However, if the metagenomic entity is present in low abundance within a subpopulation of the samples, the model will utilize the information from the higher abundance samples to optimize the signature gene set. If the signature genes are truly specific for the species, they facilitate quantification even at a very low abundance.

We find that treating SG selection as a variant of the CCP allows us to identify SGs that are easier to detect uniformly across samples. We can identify genes that do not act as expected using an NB model, which allows us to replace these with genes that are more consistently found across samples. This leads to more reliable species identification and an improved abundance estimation, since abundance is less reliant in genes that are not likely to be present or seems to be oversampled in a majority of samples. When tested on the simulated data set, the refined SG sets were found to significantly improve the relative abundance estimates compared with the initial SG sets. The MSE between the distribution that one would expect from the model was reduced for approximately 52% of all sets of signature genes. The number of SGs identified in samples with significant (*P *<* *0.05, CCP) depletion in SGs changed between pre- and post-refinement, rejecting fewer samples that otherwise showed a large representation of SGs, while still rejecting samples that had very few SGs in a given sample, which indicates the selection of a set of SGs that are more likely to be identified in unison. From the real dataset, it was clear that even in cases of low abundance species, the method was able to identify a set of SGs that are found more consistently across the samples.

## Supplementary Material

vbad060_Supplementary_Data
