# Supplementary material for: Identification of representative species-specific genes for abundance measurements
Source: Bioinform Adv. 2023 May 8;3(1):vbad060. doi: 10.1093/bioadv/vbad060 (PMC10199311; doi:10.1093/bioadv/vbad060)
Supplement: vbad060_Supplementary_Data [file vbad060_supplementary_data.doc]

**Supplementary Data** for Identification of representative species-specific genes for abundance measurements

Trine Zachariasen, Anders Østergaard Petersen, Asker Brejnrod, Aron Eklund, Gisle Alberg Vestergaard and Henrik Bjørn Nielsen

Table of Contents

[Supplementary Figure 1 2](#_Toc132362404)

[Supplementary Figure 2 3](#_Toc132362405)

[Supplementary Figure 3 4](#_Toc132362406)

[Supplementary Figure 4 5](#_Toc132362407)

[Supplementary Figure 5: 6](#_Toc132362408)

[Supplementary Figure 6 7](#_Toc132362409)


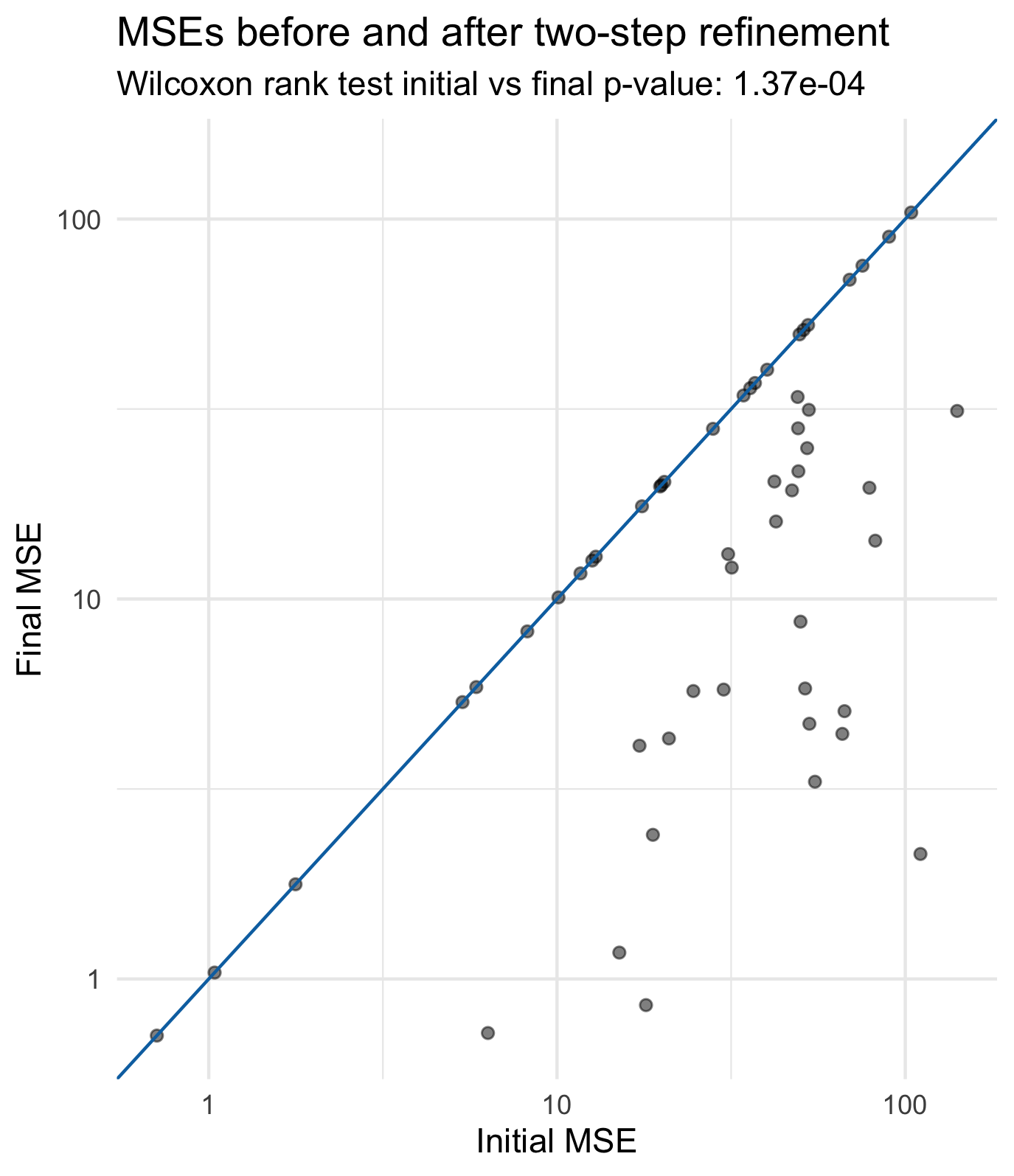


##### Supplementary Figure 1

**Improvement in MSE from initial to refined SG sets.** MSEs of the number of identified SGs, *d*, for a given number of reads assigned to SGs, *k*, before and after a two-step refinement of all 54 signature gene sets.


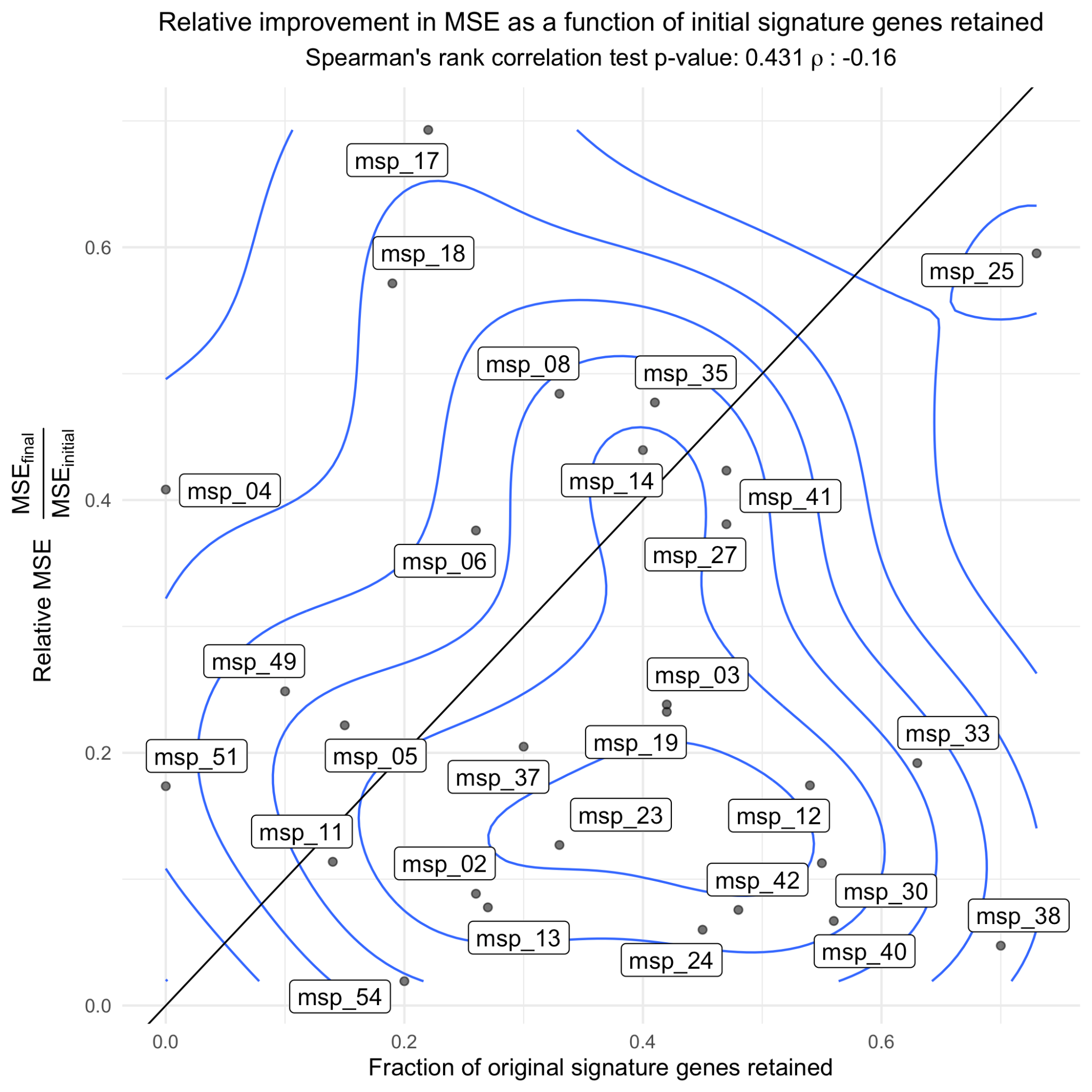


##### Supplementary Figure 2

**Relative improvement in MSE as function of initial SG’s retained**. Illustrating relative MSEs and fraction of original signature genes kept throughout refinement. Each dot is a set of SGs that has undergone SG refinement. 26 of the MSPs are not having any SGs replaced and are thus not included in the figure.


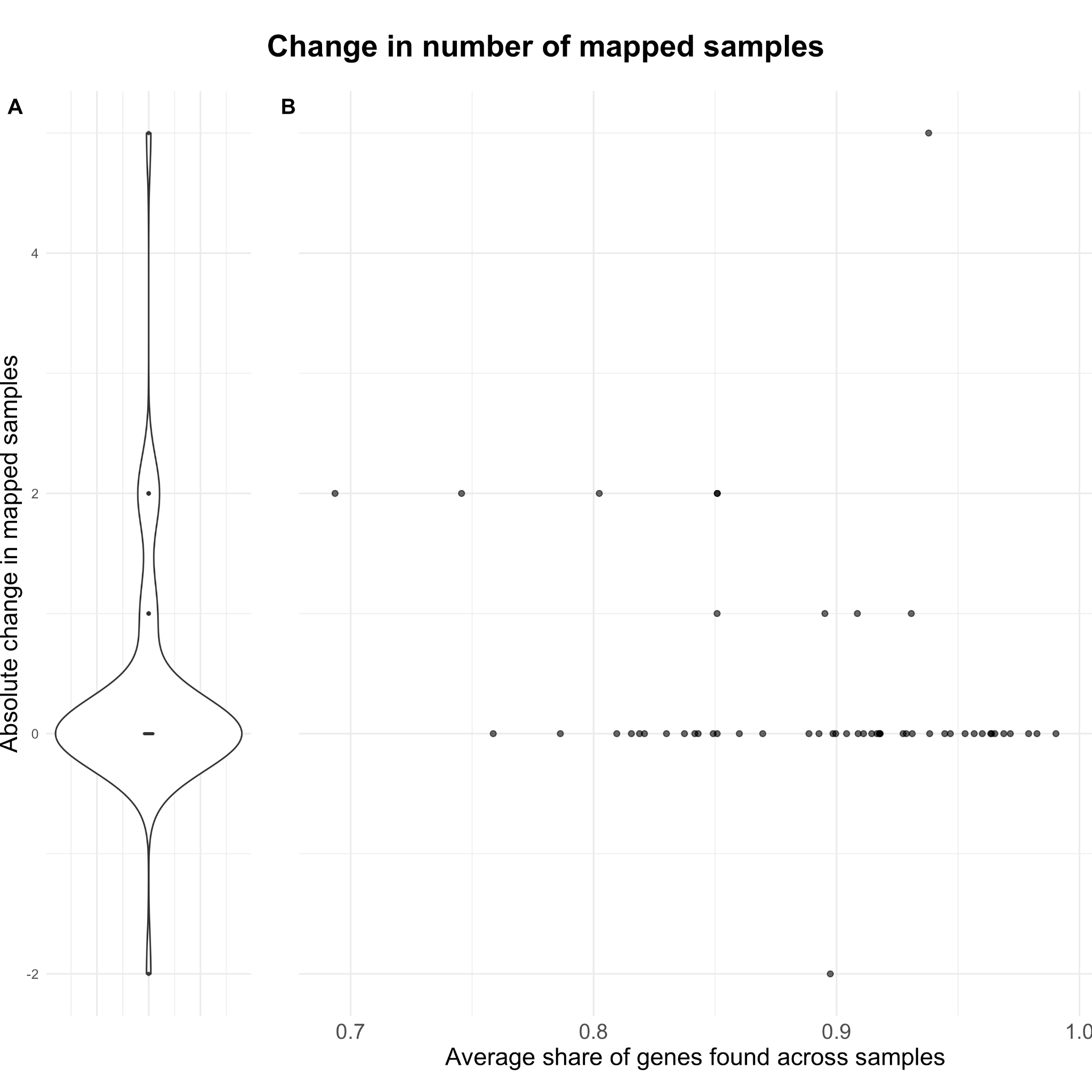


#####

##### Supplementary Figure 3:

**Change in number of mapped samples** A) Combination violin and boxplot of relative number of mapped samples for all MSPs. B) Relative change in the number of mapped samples on the y-axis and degree of overlap between initial and final set of signature genes. A sample is considered mapped if 3 or more total read counts to SGs are observed.


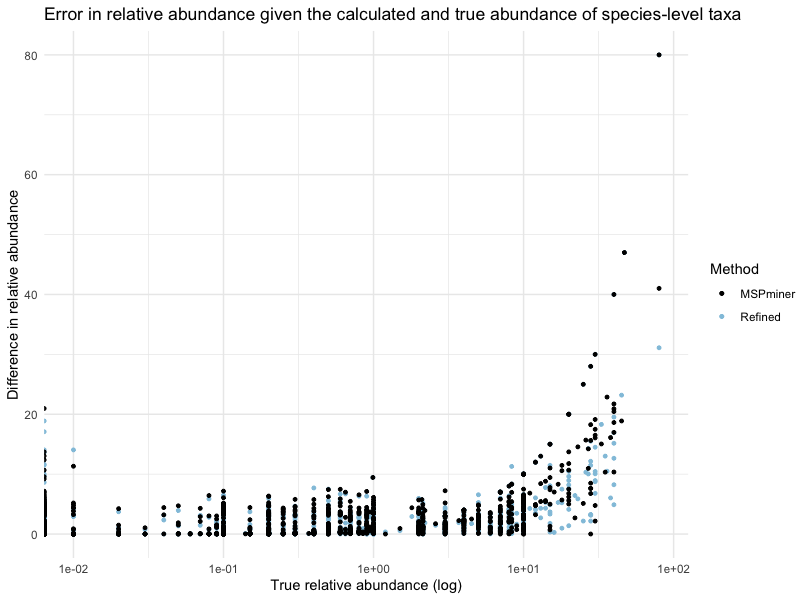


##### Supplementary Figure 4

**Error in relative abundance given the calculated and true abundance of species-level taxa.** The error of the calculated relative abundances given the true relative abundance for both the SGs from MSPminer and the refined set. The error is calculated as the absolute difference from the predicted relative abundance and the true relative abundance. Each dot represents a species-level taxa in a sample.

##### Supplementary Figure 5

**Heatmap of the initial 100 Signature Genes from Cluster5004**. Each row is a gene, and each column is a sample. The gene detection is binary (present/absent) in each sample. The samples and genes are clustered using hierarchical clustering.

##### Supplementary Figure 6

**PCoA plots of beta diversity of the First Year of Life study** using the relative abundance from the Signature Genes. PCoA was calculated with Bray-Curtis distances.
